# Higher temperatures and lower annual rainfall do not restrict, directly or indirectly, the mycorrhizal colonization of barley (*Hordeum vulgare* L.) under rainfed conditions

**DOI:** 10.1371/journal.pone.0241794

**Published:** 2020-11-05

**Authors:** Maroua Jerbi, Sonia Labidi, Anissa Lounès-Hadj Sahraoui, Hatem Chaar, Faysal Ben Jeddi

**Affiliations:** 1 Laboratoire des Sciences Horticoles LR13AGR01, Université de Carthage, Institut National Agronomique de Tunisie, Tunis, Mahrajène, Tunisia; 2 Université du Littoral Côte d′Opale, Unité de Chimie Environnementale et Interactions sur le Vivant (UCEIV), SFR Condorcet FR CNRS 3417, Calais, France; 3 Laboratoire des Grandes Cultures LR16INRAT02, Université de Carthage, Institut National Agronomique de Tunisie, Tunis, Mahrajène, Tunisia; Institut Agro - AGROCAMPUS OUEST, FRANCE

## Abstract

Whereas the role of arbuscular mycorrhizal fungi (AMF) in plant growth improvement has been well described in agroecosystems, little is known about the effect of environmental factors on AMF root colonization status of barley, the fourth most important cereal crop all over the world. In order to understand the influence of environmental factors, such as climatic and soil physico-chemical properties, on the spontaneous mycorrhizal ability of barley (*Hordeum vulgare* L.), a field investigation was conducted in 31 different sites in sub-humid, upper and middle semi-arid areas of Northern Tunisia. Mycorrhizal root colonization of *H*. *vulgare* varied considerably among sites. Principal component analysis showed that barley mycorrhization is influenced by both climatic and edaphic factors. A partial least square structural equation modelling (PLS-SEM) revealed that 39% (R²) of the total variation in AMF mycorrhizal rate of barley roots was mainly explained by chemical soil properties and climatic characteristics. Whereas barley root mycorrhizal rates were inversely correlated with soil organic nitrogen (ON), available phosphorus amounts (P), altitude (Z), average annual rainfall (AAR), they were directly correlated with soil pH and temperature. Our results indicated that AMF root colonization of barley was strongly related to climatic characteristics than chemical soil properties. The current study highlights the importance of the PLS-SEM to understand the interactions between climate, soil properties and AMF symbiosis of barley in field conditions.

## Introduction

Global climate models identified the Mediterranean region as one of the most vulnerable area to climate change [1]. In many Mediterranean countries, particularly in Tunisia, climate change projections indicate increased drought periods and a drop in rainfall (by 4 to 25%) [2, 3]. The agriculture sector in Tunisia that contributed approximately to 10.24% of the gross domestic national product in 2015 is therefore threatened by climate change. The cereals, especially wheat and barley, are produced mainly under rainfed conditions and represent 18% of the agricultural production [4]. Climate change could significantly affect the production of these crops, which are significantly important for the socioeconomic development and the stability of the country.

Barley (*Hordeum vulgare* L.) is the fourth most important cereal crop worldwide. Global barley consumption in 2018 has been estimated at 139.59 million tons [5]. In Tunisia, barley is the second cereal cultivated in all the regions of the country and occupied 0.52 million hectares [6]. Barley is considered among cereals that are well adapted to different climatic conditions [7]. However, extreme environments can negatively affect its growth and productivity [8]. In the Northern area of the country, barley is commonly grown on marginal soils under rainfed conditions (0.49 million hectares) [6]. This area has a typical Mediterranean climate, characterized by dry summers with high temperatures and mild wet winters [9, 10].

Under such environmental conditions, some beneficial microorganisms such as Arbuscular Mycorrhizal Fungi (AMF) have been proven to provide many benefits to their host plants by (i) improving water and mineral nutrient uptake [11], (ii) increasing tolerance to biotic and abiotic stresses [12–16] and (iii) enhancing soil aggregation stability [17, 18]. In general, the frequency of AMF in roots is higher when there is not enough water for the plant to grow and survive [19].

Radical colonization by AMF is also constrained by climatic variables. According to Zhang et al. [20], mycorrhizal colonization was directly correlated with precipitation. In contrast, Augé et al. [21] found that AMF colonization increased under water limiting conditions. Regarding the temperature effect, it has been demonstrated that AMF colonization was higher at 20°C than at 12°C [22]. However, the responses of AMF to an increase or a decrease in temperature seem to vary according to the host plant species [23].

The establishment of the symbiosis could be influenced by soil properties such as texture [24], pH [25], lime (CaCO_3_) [26], organic matter content [27] and minerals such as nitrogen and phosphorus, and some of these are modified by an increase in temperature [28] and a decrease in soil moisture [29], as a result of a scant rainfall. The combined effect of physico-chemical soil characteristics on AMF root colonization of cereals, in particular barley, is not yet clearly elucidated. Presently, only a few studies have reported the combined effect of soil parameters and climate on AMF symbiosis [30–32].

To the best of our knowledge, spontaneous mycorrhizal colonization of cereal crops, in particular barley, has not yet been investigated in Tunisia. No data are available concerning the effects of edaphic and climatic factors on natural AMF colonization of barley. Therefore, the present work aims to analyze the effect of interactions between different groups of environmental factors (climate and soil physico-chemical properties) on AMF colonization status of barley (*Hordeum vulgare* L.), through the use of a Partial Least Square Structural Equation Modeling (PLS-SEM) method.

## Materials and methods

### Ethic statement

The study was not conducted in protected areas. The 31 sites were located in private agricultural fields. We obtained verbal agreements from owners and we did not need any official paper. We thank the farmers for their cooperation in this research. We did not need any official permission from authorities because we had contacted the farmers directly. They gave us their permission to take soil and plant samples from their lands without any problem.

We confirm that the field study was not carried out in a protected area and did not involve any threatened and endangered species.

### Field site description

**Fig 1:**
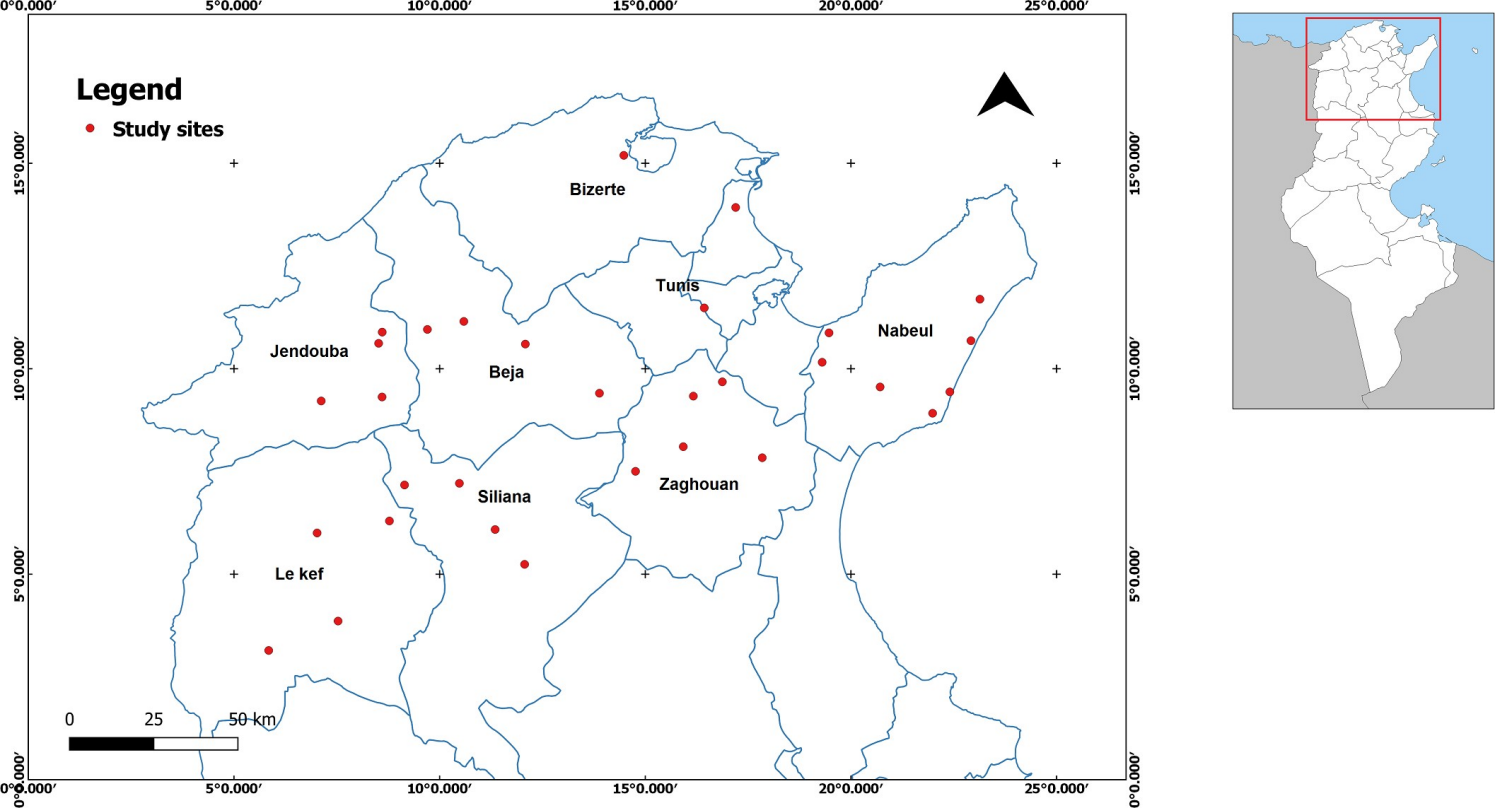
Geographic locations of the 31 sampling sites in Northern Tunisia.

The present study was carried out in Northern Tunisia where cereal crops are mainly cultivated under rainfed conditions. In this area, 31 sites of barley (*Hordeum vulgare* L.) were selected randomly, based on their geographic coordinates (Fig 1): latitude (*Y*), longitude (*X*) and elevation (*Z*), and their bioclimatic stages. For each site, weather conditions: the average annual rainfall (*AAR*), maximum temperature of the warmest month (*M*) and minimum temperature of the coldest month (*m*), were obtained using MRRA-2 application V5.12.4 [33] during the period of the investigation (2015–2016).

### Soil and root sampling

From each site, three replicates of roots with their rhizospheric soil (0-20cm) were collected along a diagonal transect at least 3m apart during the heading stage [BBCH 59; 34] (April 2016-May 2016). In total, 93 soil and root samples were taken. Root samples were washed in tap water, stored in ethanol (50%) and conserved at 4°C before measuring the AMF colonization. Soil samples were conserved at 4°C until the determination of physico-chemical characteristics.

### Soil analysis

Soil moisture [%] was determined with the gravimetric method using the difference between the weights of soil samples before and after drying at 105°C for 48 h. Soil texture was estimated using the Robinson’s pipette method [35]. Soil organic carbon [%] was determined with the Walkley and Black method as described by Pauwels et al. [36], organic matter in the soil was calculated by multiplying organic carbon by 1.72. The total calcium carbonates (CaCO_3_) amount was analyzed using the Bernard calcimeter method [37]. Soil samples were analyzed for pH in an (1:2.5) soil:water suspension, and soluble salts were determined by measuring the electrical conductivity (EC) [dS m^-1^] in an (1:5) soil: water suspension at 25°C [36]. Soil available phosphorus (P) [ppm] was measured according to the Olsen method [38]. Soil Organic Nitrogen (ON) [mg g^-1^] was determined using the Kjeldahl method followed by titration [39].

### AMF root colonization

To determine AMF root colonization, barley roots were colored according to the method of Phillips and Hayman [40]. Roots were cleared in KOH (2.5%), rinsed in distilled water, then acidified in HCL (1%) and stained with trypan blue (0.05%). The percentage of total mycorrhization was determined using the method of McGonigle et al. [41]. Root fragments from the 93 plants collected in all the studied sites were mounted on microscope slides and observed at (40×) and (100×) magnification, in order to count mycorrhizal structures (arbuscules, vesicles and hyphae). For each site, 225 observations (25 root fragments × 3 intersections per root fragment × 3 replicates) were examined.

### Statistical analysis

In order to satisfy the homogeneity and normality assumptions in statistical analysis, the data were examined for skewness using Shapiro-Wilk test. Available phosphorus (P) values were log converted and square root transformation was used for soil organic nitrogen (ON) amounts. The mean values and standard deviations were calculated from three replicates per site. The significant difference between mean values of mycorrhizal colonization across the different studied sites was determined with One-way Analysis of Variance (ANOVA) with Fisher’s least significant difference (LSD) test at *P* < 0.05.

Principal component analysis (PCA) was performed on soil properties, climatic characteristics and mycorrhizal colonization in the different sites. It was developed on mean centered variables. A hierarchical ascendant classification (HAC) on principal components was then performed using Euclidean distances to measure similarity among sites.

Next, Pearson’s correlation analysis was conducted to elucidate the relationship between environmental factors and AMF colonization. These statistical analyses were performed using R software [version 3.6.0; 42].

Partial least squares structural equation model (PLS-SEM) was applied to test the importance and significance effects of environmental factors on mycorrhizal colonization of barley. This method has less restrictive assumptions for data normality and is used to model highly complex relationships between independent and dependent variables by utilizing a multiple regression approach. One of the advantages of this technique is the use of unmeasured variables as latent variables estimated from measured variables (manifest variables) in the model [43]. Physico-chemical soil properties and site characteristics were used as predictor variables and AMF root colonization as the response variable. Each latent variable was composed of a block of manifest variables (Table 1). Multi-collinearity between manifest variables was measured by evaluating the Variance Inflation Factor (VIF) [44]. SmartPLS 3.2.8 Pro software [45] was used to design the model and to determine path coefficients, coefficient of determination (*R²*), which represent the model’s predictive accuracy [46], and the significance of the weights and loadings of manifest variables. The Cross-validated redundancy (*Q*²) was used to assess the model’s predictive relevance [47]. The relevance of the latent variables was also examined using the effect size (*f²*) and its guidelines developed by Cohen [48].

**Table 1:** Descriptive statistics of soil properties, climatic characteristics and AMF root colonization; Standard Deviation (STDEV), Range [min, max], *n* = 93 (31 sites with 3 replicates/site).

Latent variables: (unmeasured variables)	Manifest variables (measured variables)	Abbreviation	Mean ± STDEV	Range
Predictor variables				
**1. Chemical soil properties**	pH (H_2_O)	pH	7.72 ± 0.38	[6.99, 8.6]

	Soil salinity [dS m^-1^]	Salinity	0.17 ± 49.30	[0.06, 0.32]

	Total calcium carbonate [%]	Total CaCO_3_	29.52 ± 19.05	[2, 68.5]

	Organic carbon content [%]	OC	1 ± 0.37	[0.3, 1.9]

	Organic nitrogen [mg g^-1^]	ON	0.64 ± 0.41	[0.32, 1.48]

	Available phosphorus [ppm]	P	51.88 ± 17.72	[18.7, 107]

**2. Physical soil properties**	Soil clay content [%]	SCC	37.74 ± 14.79	[6.9, 61.5]

	Soil sand content [%]	SSC	30.19 ± 16.57	[2.3, 67.5]

	Soil moisture content [%]	SMC	12.39 ± 5.23	[4.2, 24.8]

**3. Site climate**	Average annual rainfall [mm]	AAR	413.19 ± 65.14	[323.9, 508.2]

	Maximum temperature of the warmest month [°C]	M	27.67± 0.82	[26.2, 29.3]

	Minimum temperature of the coldest month [°C]	m	8.68 ± 1.9	[5.3, 11.9]

	Altitude [m a.s.l]	Z	215.6 ± 193.53	[2, 649]
**Response variable**				
**AMF root colonization**	Total AMF colonization rate [%]		27.2 ± 14.65	[5.6, 75.6]

## Results

### AMF root colonization of Barley

In the present research, the studied sites had significantly different levels of AMF root colonization (Table 2). One-way ANOVA showed that total colonization rates were significantly different between sampling sites (*F* = 38.7, *P* < 0.001, S1 Table). The maximum value of total mycorrhizal rate was recorded at “*Charfine*” (67.8%), whereas the lowest value (8.5%) was observed at “*Balta*” and “*Bourada*” (Table 2). According to the one-way ANOVA, arbuscular colonization was significantly affected by sites (*F* = 35.47, *P* < 0.001, S1 Table). Arbuscules were observed in all collected roots. Arbuscular colonization was significantly greater at “*Charfine*” (58%) and “*Zaghouan*” (56.3%) compared with the other sites (*F* = 35.47, *P* < 0.001). Vesicle colonization was significantly different across the different sites (*F* = 6.11, *P* < 0.001, S1 Table). In fact, the colonization rate of vesicles were significantly higher at “*Dougga*” (11.8%), “*SidiDaher*” (10.4%) and “*Charfine*” (9.6%) than at the other sites. Vesicles were observed only in barley roots originating from 13 sites.

**Table 2:** Mycorrhizal colonization in *H*. *vulgare* roots at the 31 sampling sites.

Studied sites	Mycorrhizal rate (%)	Arbuscules (%)	Vesicles (%)
Charfine	67.8 ± 7.80 **a**	57.8 ± 5.9 **a**	9.6 ± 5.5 **a**
Zaghouan	56.7 ± 4.0 **b**	56.3 ± 3.4 **a**	5.6 ± 5.6 **b**
Morneguia	48.8 ± 5.6 **c**	46.3 ± 3.6 **b**	2.8 ± 4.2 **bcd**
Elmida	48.2 ± 1.3 **c**	47.1 ± 1.7 **b**	3.3 ± 3.4 **bcd**
Birmchergua	44.8 ± 7.4 **cd**	43.7 ± 8.6 **bc**	1.5 ± 1.7 **cd**
Soliman	38.9 ± 2.9 **de**	38.9 ± 2.9 **cd**	0.0 ± 0.0 **d**
SidiDaher	38.9 ± 5.1 **de**	34.1 ± 3.4 **d**	10.4 ± 2.5 **a**
Goubellat	38.9 ± 3.3 **de**	38.9 ± 3.3 **cd**	0.0 ± 0.0 **d**
Tazarka	35.2 ± 3.2 **e**	35.2 ± 3.2 **d**	1.5 ± 1.7 **cd**
Oued Zarga	34.1 ± 3.4 **e**	34.1 ± 3.4 **d**	0.0 ± 0.0 **d**
Souala	27.0 ± 6.7 **f**	27.0 ± 6.7 **e**	4.4 ± 5.1 **bc**
BeniKhiar	26.3 ± 5.5 **f**	26.3 ± 5.5 **e**	0.0 ± 0.0 **d**
GalaatAndalous	25.9 ± 1.7 **fg**	25.9 ± 1.7 **ef**	0.0 ± 0.0 **d**
Benouria	25.9 ± 5.3 **fg**	25.9 ± 5.3 **ef**	4.4 ± 5.1 **bc**
Skhira	23.3 ± 1.1 **fgh**	23.3 ± 1.1 **efg**	0.0 ± 0.0 **d**
Mneguaa	21.8 ± 3.5 **fghi**	21.8 ± 3.5 **efgh**	0.0 ± 0.0 **d**
Bargou	20.7 ± 2.8 **fghi**	20.7 ± 2.8 **efgh**	0.0 ± 0.0 **d**
Jaddara	20.7 ± 2.3 **fghi**	20.7 ± 2.3 **efgh**	1.8 ± 1.7 **bcd**
SidiHassen	20.7 ± 20.7 **fghi**	20.7 ± 20.7 **efgh**	0.0 ± 0.0 **d**
Menzeltemim	19.6 ± 4.5 **ghij**	19.6 ± 4.5 **fghi**	0.0 ± 0.0 **d**
Dougga	19.6 ± 2.8 **ghij**	19.6 ± 2.8 **fghi**	11.8 ± 2.8 **a**
Dahmeni	19.3 ± 2.5 **hji**	19.3 ± 2.5 **ghi**	1.8 ± 2.3 **bcd**
Kef	17.4 ± 2.8 **hijk**	17.4 ± 2.8 **ghij**	0.0 ± 0.0 **d**
Sers	17.0 ± 2.4 **hijk**	17.0 ± 2.4 **ghij**	5.6 ± 1.2 **b**
Mhamid	17.0 ± 3.4 **hijk**	17.0 ± 3.4 **ghij**	0.0 ± 0.0 **d**
Ghiriya	15.9 ± 2.4 **ijkl**	15.9 ± 2.4 **hijk**	0.0 ± 0.0 **d**
Zribahammem	14.1 ± 5.6 **jklm**	14.1 ± 5.6 **ijkl**	0.0 ± 0.0 **d**
Kodiya	11.8 ± 5.1 **klm**	11.8 ± 5.1 **jkl**	0.0 ± 0.0 **d**
OuedBeja	10.0 ± 2.9 **lm**	10.0 ± 2.9 **kl**	0.0 ± 0.0 **d**
Balta	8.5 ± 2.8 **m**	8.5 ± 2.8 **l**	0.0 ± 0.0 **d**
Bouarada	8.5 ± 1.7 **m**	8.5 ± 1.7 **l**	0.0 ± 0.0 **d**

Data is presented as mean ± standard deviation. Means were obtained from three replicates per site. Different letters in the same column indicate significant differences at P < 0.05 (Fisher’s LSD test).

### Environmental factors and AMF colonization

**Fig 2:**
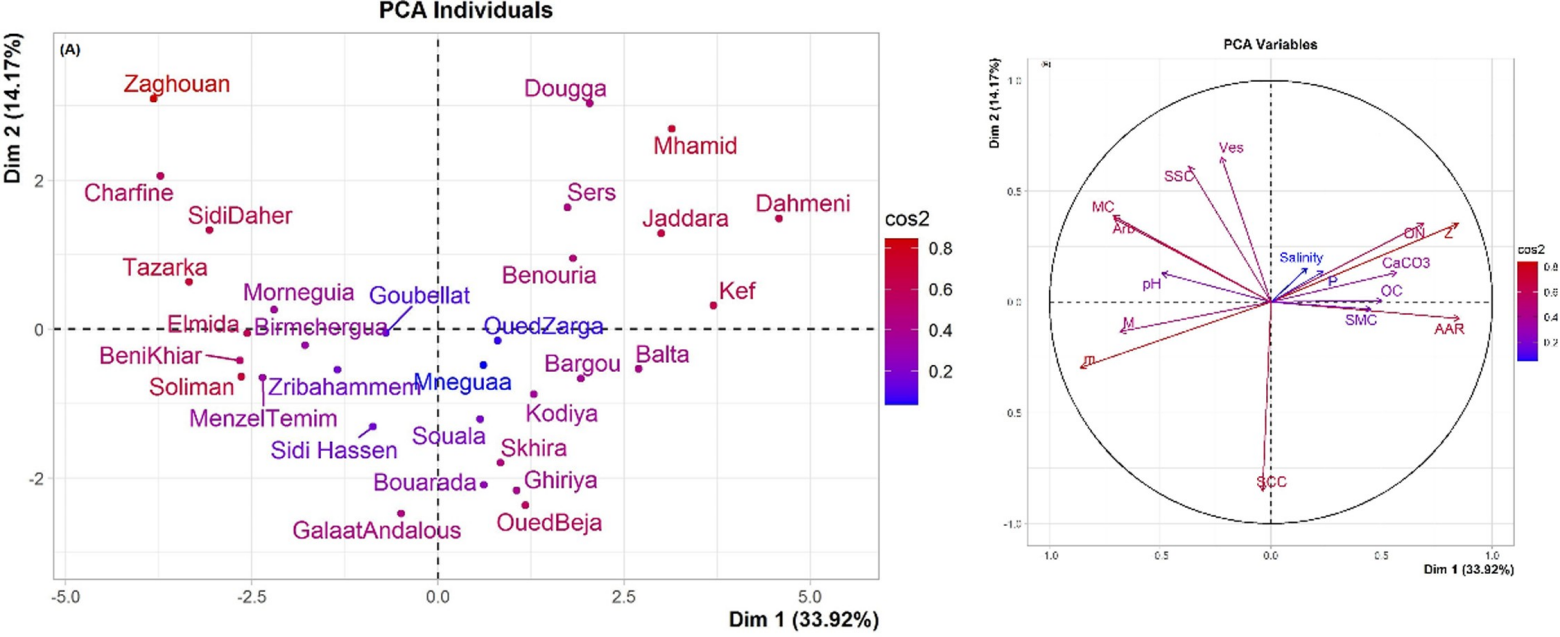
Principal components analysis (PCA) between environmental variables (soil physico-chemical properties and climatic characteristics) and mycorrhizal colonization in the 31 sampling sites of Northern Tunisia (with the mean values).

According to the results of PCA analysis at the different sites, the first two components (PC1 and PC2) together explained 48.09% of variability in the dataset (Fig 2) (total inertia decomposition is shown in S1 Fig). The first principal component (PC1) which accounted for 33.92% of the variance, was strongly and directly correlated with AAR (r = 0.85, *P* < 0.0001), Z (r = 0.85, *P* < 0.0001) and ON (r = 0.69, *P* < 0.0001). On the other hand, m (r = -0.86, *P* < 0.0001), M (r = -0.68, *P* < 0.0001) and MC (r = -0.71, *P* < 0.0001) showed a high and inverse effect on PC1 (Table 3). The second principal component (PC2) explained 14.17% of the total variation and had the highest positive values for SSC (r = 0.61, *P* < 0.001) and Ves (r = 0.65, *P* < 0.0001). However, SCC (r = -0.85, P < 0.0001) was inversely correlated with PC2. PCA revealed that the sites “*Mhamid*,” “*Jaddara”*, “*Kef*”, “*Dahmeni*”, “*Dougga”* and “*Sers”* were different from “*Zaghouan”*, “*Tazarka”*, “*Soliman”*, “*SidiDaher”*, “*Charfine”* and “*BeniKhiar”* along PC1 by its high (Z, AAR and ON) and its low (m, M and MC). Also, the variables (SCC, SSC and Ves) contributed to separate the sites “*OuedBeja*”, “*Skhira*” and “*GalaatAndalous*” from the others along PC2. Similar results were obtained when examining the contribution of each variable to the total variance explained by the PCA axes.

Sub figures show the variation in sites scores (A) and variables scores (B) along the first two PCA axes. 33.92 and 14.17% of the variation are explained by PC1 (Dim. 1) and PC2 (Dim.2), respectively. The lengths of the arrows indicate the relative importance of each variable, whereas the angles between the arrows indicate the degree to which they are correlated. Salinity, soil salinity; OC, soil organic carbon; ON, soil organic nitrogen; P, available P; CaCO_3_, total calcium carbonates content; SCC, soil clay content; SSC, soil sand content; SMC, soil moisture content; AAR, average annual rainfall; M, maximum temperature of the warmest month; m, minimum temperature of the coldest month; Z, altitude; MC, total Mycorrhizal Colonization; Arb, Arbuscular colonization; Ves, Vesicle colonization.

**Table 3:** Correlations between soil properties, climatic characteristics, mycorrhizal colonization of *H*. *vulgare* and PCA axes.

Variable	PC1	PC2	PC3
pH	-0.49^*^	0.13	-0.06
Salinity	0.16	0.15	0.54^*^
OC	0.50^*^	0.01	0.53^*^
ON	0.69^***^	0.35	0.05
P	0.24	0.14	-0.57^**^
CaCO_3_	0.57^**^	0.13	0.58^**^
SCC	-0.04	-0.85^***^	0.20
SSC	-0.37^(^^.^^)^	0.61^**^	-0.43^(^^.^^)^
SMC	0.45^(^^.^^)^	-0.03	0.27
AAR	0.85^***^	-0.07	-0.05
M	-0.68^***^	-0.13	0.21
m	-0.86^***^	-0.30	-0.06
Z	0.85^***^	0.36^(^^.^^)^	-0.10
MC	-0.71^***^	0.39^(^^.^^)^	0.48^*^
Arb	-0.71^***^	0.38^(.)^	0.47^*^
Ves	-0.22	0.65^***^	0.03

Significance level: ‘.’correlation is significant at P < 0.05.

‘*’correlation is significant at P < 0.01.

‘**’correlation is significant at P < 0.001.

‘***’correlation is significant at P < 0.0001.

**Fig 3:**
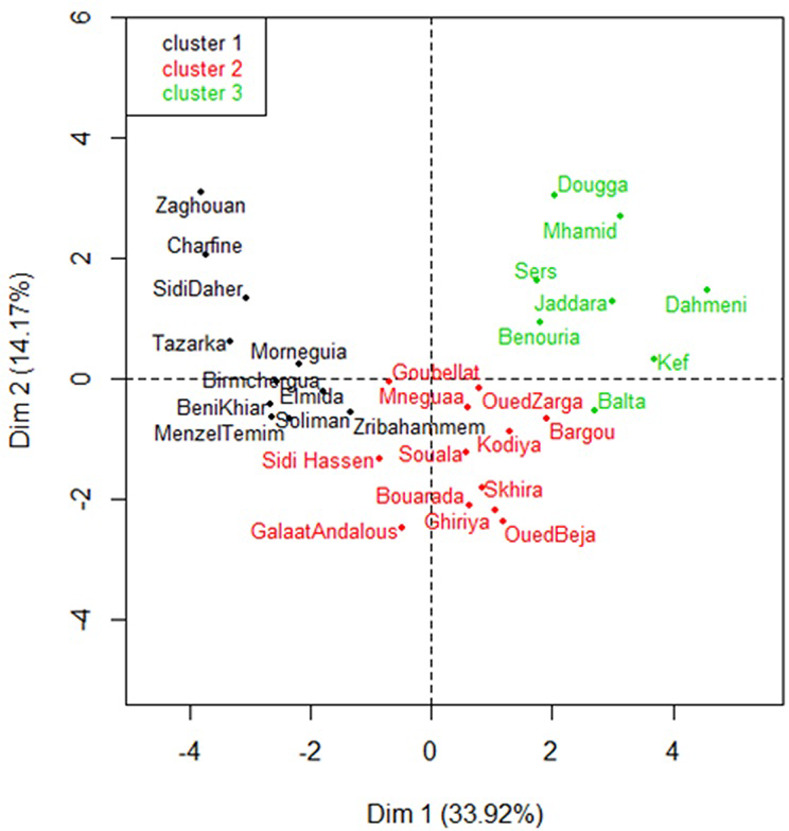
Hierarchical Ascendant Classification (HAC) on principal component analysis (PCA) of the studied sites. Dim.1 and Dim. 2 are the two first dimensions issued from the PCA.

The HCA approach revealed three clusters according to their soil physico-chemical properties, climatic characteristics and mycorrhizal rates. The resulting factor map is shown in Fig 3 and the relative matrix with quantitative dissimilarities and HCA dendrogram are shown in S2 Table and S2 Fig, respectively. The sites combined in cluster 1 are characterized by the highest m, M, SSC, MC and Arb values, associated with the lowest AAR, Z and ON values, whereas sites in cluster 3 were characterized by the opposite. Cluster 2 was described by sites with the greatest SCC values. These results were in accordance with results from the PCA.

**Fig 4:**
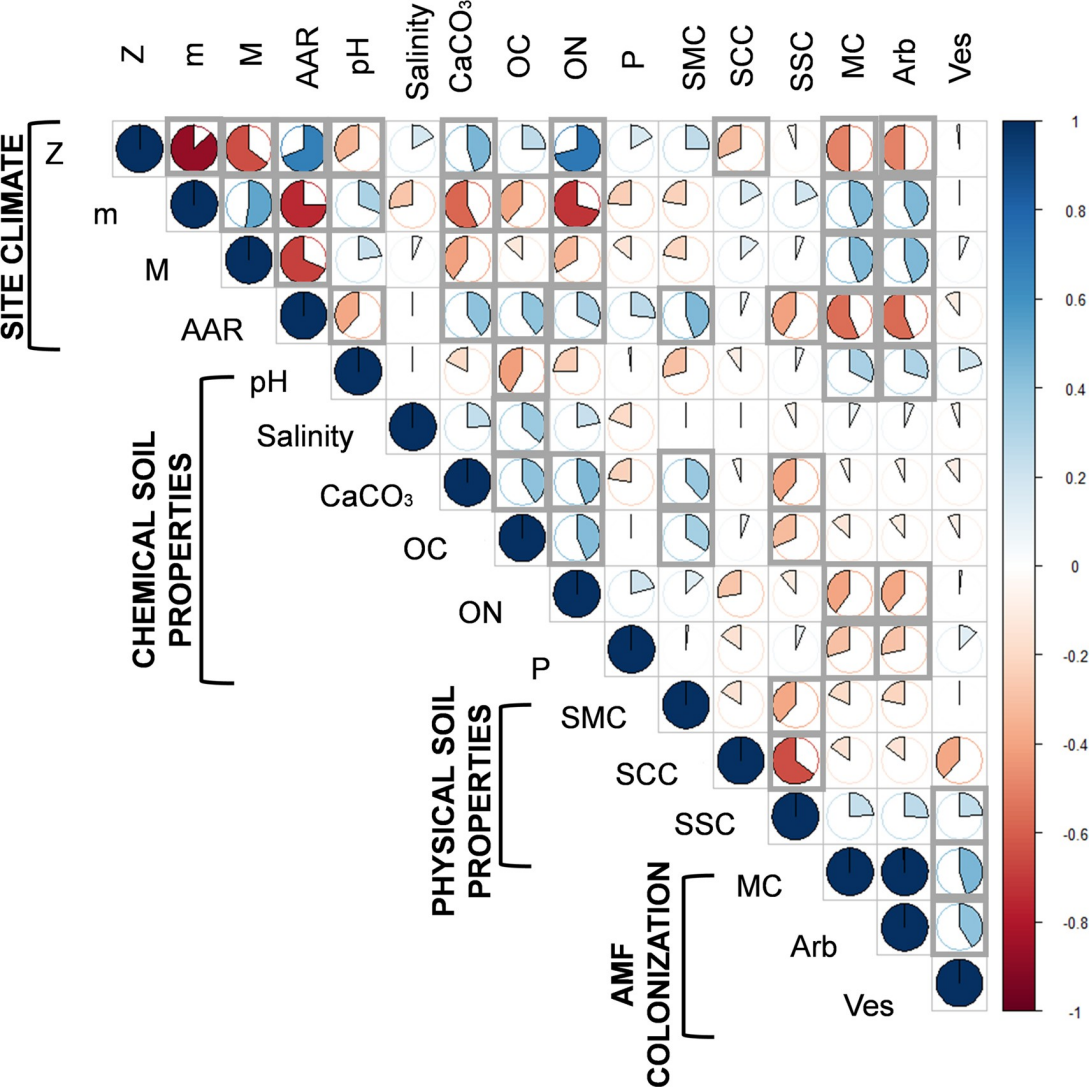
Pearson’s correlation matrix between environmental factors and AMF root colonization. Salinity, soil salinity; OC, soil organic carbon; ON, soil organic nitrogen; P, available P; CaCO_3_, total calcium carbonates content; SCC, soil clay content; SSC, soil sand content; SMC, soil moisture content; AAR, average annual rainfall; M, maximum temperature of the warmest month; m, minimum temperature of the coldest month; Z, altitude; MC, total Mycorrhizal colonization, Arb, Arbuscular colonization; Ves, Vesicle colonization.

The Pearson’s correlation analysis was used to assess the relationship between AMF colonization and all environmental variables (Fig 4 and Table 4). In this study, the site’s climatic factors were the most important explanatory variables (Fig 4). The results indicated that mycorrhizal colonization of barley was highly and inversely correlated with ON (r = -0.39, *P* < 0.05), AAR (r = -0.56; *P* < 0.001) and Z (r = -0.49; *P* < 0.001). Strong and direct correlations existed between AMF root colonization, M and m (r = 0.45, *P* < 0.05). There were no significant relationships between Salinity, OC, total CaCO_3_, SCC and AMF root colonization. Mycorrhizal colonization of barley at the different sites was mainly affected by the climatic variables (m, M, AAR and Z). These results corroborate those obtained with PCA and HCA analyses.

**Table 4:** Pearson's correlation analysis showing correlations (*R* values) between environmental variables and AMF root colonization.

Variable	pH	Salinity	OC	ON	P	CaCO_3_	SCC	SSC	SMC	AAR	M	m	Z	MC	Arb	Ves
**pH**	1															
**Salinity**	0.00	1														
**OC**	-0.41^*^	0.37^*^	1													
**ON**	-0.25	0.23	0.43^*^	1												
**P**	-0.02	-0.19	0.01	0.21	1											
**CaCO**_**3**_	-0.18	0.25	0.41^*^	0.44^*^	-0.23	1										
**SCC**	-0.10	0.00	0.05	-0.28	-0.15	-0.05	1									
**SSC**	0.06	-0.07	-0.31^(.)^	-0.11	0.07	-0.40^*^	-0.65^***^	1								
**SMC**	-0.30	-0.01	0.34^(.)^	0.13	0.01	0.38^*^	-0.15	-0.38^*^	1							
**AAR**	-0.39^*^	0.01	0.39^*^	0.33.	0.27	0.40^*^	0.06	-0.41^*^	0.45^*^	1						
**M**	0.23	0.06	-0.13	-0.33.	-0.14	-0.40^*^	0.13	0.06	-0.22	-0.69^***^	1					
**m**	0.31^(^^.^^**)**^	-0.27	-0.39^*^	-0.71^***^	-0.24	-0.58^***^	0.17	0.19	-0.23	-0.75^***^	0.52^***^	1				
**Z**	-0.35^(**.)**^	0.17	0.25	0.71^***^	0.17	0.45^**^	-0.32^(.)^	-0.06	0.25	0.69^***^	-0.65^***^	-0.87^***^	1			
**MC**	0.32^(**.)**^	0.07	-0.13	-0.39^*^	-0.30^(**.)**^	-0.07	-0.15	0.24^(.)^	-0.19	-0.56^***^	0.45^*^	0.45^*^	-0.49^***^	1		
**Arb**	0.30^(**.)**^	0.06	-0.11	-0.39^*^	-0.29^(**.)**^	-0.07	-0.14	0.26	-0.22	-0.56^***^	0.45^*^	0.44^*^	-0.50^***^	0.99^***^	1	
**Ves**	0.20	-0.05	-0.08	0.01	0.13	-0.10	-0.38^*^	0.25	0.00	-0.10	0.06	0.00	-0.01	0.45^*^	0.41^*^	1

Salinity, soil salinity; OC, soil organic carbon; ON, soil organic nitrogen; P, available P; CaCO_3_, total calcium carbonates content; SCC, soil clay content; SSC, soil sand content; SMC, soil moisture content; AAR, average annual rainfall; M, maximum temperature of the warmest month; m, minimum temperature of the coldest month; Z, altitude; MC, total Mycorrhizal colonization, Arb, Arbuscular colonization; Ves, Vesicle colonization. Significant *R* values are written in bold font. Significance level: ‘.’correlation is significant at P < 0.1.

‘*’correlation is significant at P < 0.05.

‘**’correlation is significant at P < 0.01.

‘***’correlation is significant at P < 0.001.

PLS-SEM was developed to further examine the causal effects of these environmental factors on AMF colonization of barley. Two manifest variables: m and total CaCO_3_ contents in the soil, were discarded from the final path model because they exhibited very high VIF values (> 5) (S3 Table). All retained manifest variables and latent variables were free of multicollinearity, with VIF values varying between 1.000 and 2.935. PLS-SEM was used to test if the effect of site climate on AMF colonization of barley could be mediated by indirect interactions with soil physico-chemical properties. However, because of weak and non-significant interactions, these indirect effects canceled out of the final path model. Thus, the total effect of site climate was driven by only the direct effects.

**Fig 5:**
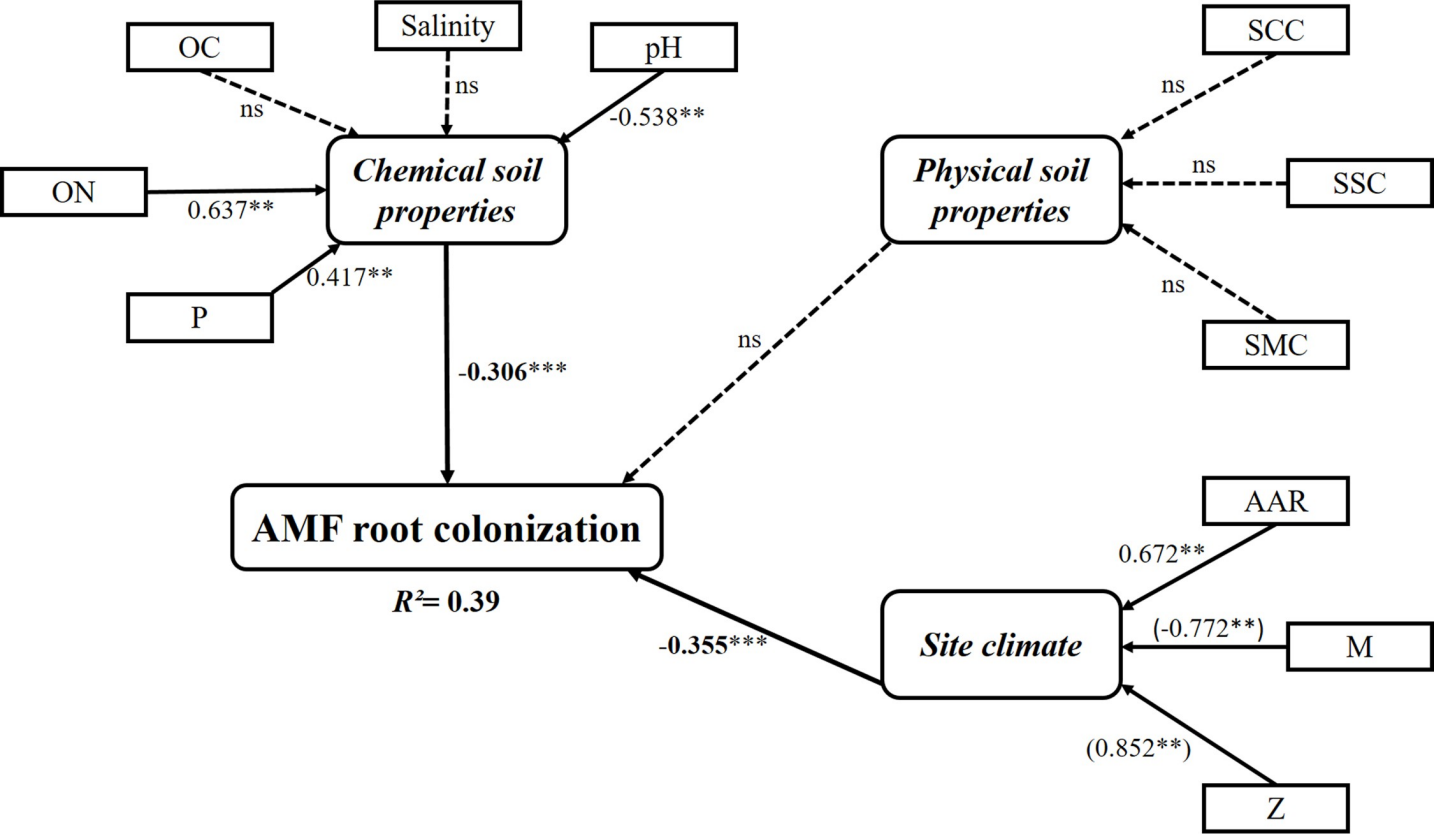
Partial least square structural equation model (PLS-SEM) showing the relationships among soil physico-chemical properties, climatic characteristics and AMF colonization. R² = 39% and Q² = 29.1%. Solid lines and dashed lines indicate significant and non-significant pathways, respectively. The numbers near the arrows indicate the standardized weights or (loadings) and path coefficients (*correlation is significant at P < 0.1, **correlation is significant at P < 0.05, ***correlation is significant at P < 0.01). Salinity, soil salinity; OC, soil organic carbon; ON, soil organic nitrogen; P, available P; SCC, soil clay content; SSC, soil sand content; SMC, soil moisture content; AAR, average annual rainfall; M, maximum temperature of the warmest month; Z, altitude.

The final model representing the weights, the path coefficients, and the coefficient of determination (R²) is shown in Fig 5. PLS path model revealed that chemical soil properties and site climate explained a moderate variance (*R²* = 0.39) of the total AMF root colonization with a predictive relevance (*Q²* = 0.29). This model suggested that chemical soil properties (-0.31 path coefficient) and site climate (-0.36 path coefficient) with small effect sizes (*f²*) of 0.10 and 0.13, were the main latent variables that influence AMF root colonization of barley (Table 5). According to the PLS-path model, mycorrhizal colonization of barley was more related to climatic variables (AAR, M and Z) (Table 5). On the other hand, chemical soil properties (ON, pH and P) also had an influence on this symbiosis (Fig 5 and Table 5). Both pH and M had a direct and significant effect on the percentage of AMF colonization in barley (—0.54 weight for pH and—0.77 loading for M) (Table 5). However, ON (-0.64 weight), P (-0.42 weight), AAR (-0.67 weight) and Z (-0.85 loading) all had a significant and inverse effect.

**Table 5:** PLS path model showing the weight and loading values, the path coefficients, its standard deviation, its significance relevance with *t*-values and its Bias Corrected Confidence Interval (95%).

	**Weight**	**Loading**	**Standard deviation of weight**	**Standard deviation of loading**	***t*-value of weight**	***t*-value of loading**	**95% Confidence Interval (Bias corrected) of weight**	**95% Confidence Interval (Bias corrected) of loading**
*pH ➔ Chemical soil properties*	-0.538^**^	-0.620	0.161	0.175	3.344	3.544	[-0.823, -0.241]	[-0.858, -0.268]
*Salinity ➔ Chemical soil properties*	-0.043	-0.054^ns^	0.205	0.169	0.208	0.320	[-0.406, 0.372]	[-0.381, 0.278]
*OC ➔ Chemical soil properties*	-0.214	0.249^ns^	0.201	0.161	1.068	1.545	[-0.642, 0.143]	[-0.082, 0.533]
*ON ➔ Chemical soil properties*	0.637^**^	0.756	0.195	0.110	3.268	6.860	[0.246, 0.963]	[0.583, 0.932]
*P ➔ Chemical soil properties*	0.417^**^	0.567	0.149	0.127	2.795	4.453	[0.123, 0.698]	[0.343, 0.796]
*SCC ➔ Physical soil properties*	0.404	0.571^ns^	0.532	0.415	0.759	1.376	[-0.667, 1.337]	[-0.324, 0.992]
*SSC ➔ Physical soil properties*	-0.399	-0.891^ns^	0.746	0.591	0.535	1.508	[-1.368, 1.110]	[-1.000, -0.548]
*SMC ➔ Physical soil properties*	0.600	0.690^ns^	0.378	0.435	1.588	1.587	[-0.070, 1.336]	[-0.380, 0.986]
*AAR ➔ Site climate*	0.672^**^	0.964	0.144	0.041	4.659	23.401	[0.383, 0.942]	[0.904, 0.999]
*M ➔ Site climate*	-0.100	-0.772^**^	0.250	0.110	0.401	7.020	[-0.569, 0.408]	[-0.929, -0.515]
*Z ➔ Site climate*	0.323	0.852^**^	0.225	0.075	1.438	11.321	[-0.094, 0.775]	[0.686, 0.965]
	**Path coefficient**		**Standard deviation of Path**		***t*-value of path**		**95% Confidence Interval (Bias corrected) of path**	**Effect size (*f²*) of path**
*Chemical soil properties* ➔ AMF root colonization	-0.306^***^		0.088		3.527		[-0.451, -0.123]	**0.104**^**a**^
Physical soil properties * ➔ AMF root colonization*	-0.137^ns^		0.139		0.985		[-0.304, 0.266]	0.026^b^
*Site climate ➔ AMF root colonization*	-0.355^***^		0.094		3.782		[-0.531, -0.167]	**0.125**^**a**^

R² value: 0.390.

Significant values are written in bold font:

* P < 0.1,

** P < 0.05,

*** P < 0.01.

a = small effect size,

b = no effect.

## Discussion

### Mycorrhizal colonization

In the present study, natural mycorrhizal root colonization was significantly observed in barley roots in all the prospected field sites. The percentage of mycorrhizal root colonization, ranging between 8.5 and 67.8%, varied significantly among the 31 sites. Similarly, in a study conducted in South Australia, Grace [49] found that natural mycorrhization of barley was between 9 and 47% in different field sites. Compared to the vesicles, the high percentage of arbuscules observed in the roots of barley at the “Heading” stage could be explained by the high nutrient demand of the plants for grain filling [50]. Vesicles serve as storing organs for lipids and other energy reserves [51]. Vesicle colonisation has been associated with increased consumption of carbon for AMF maintenance and construction investments, in particular under nutrient-limiting conditions [52]. The lack of vesicles in barley roots in some sites or the very low presence in other sites could be related to the low C availability to the fungal symbiont [53]. The presence of vesicles in different percentages could also be linked to the AMF taxa that colonized the barley root. For example, species of *Gigasporaceae*, *Paraglomaceae* and *Archaeosporaceae* do not produce vesicles [54, 55], whereas, *Glomaceae* and *Acaulosporaceae* do [56].

### Mycorrhizal colonization and soil properties

Our findings show that mycorrhizal colonization of barley was significantly related to chemical soil properties. Soil pH was found to have a direct correlation with AMF root colonization. According to Dumbrell et al. [57], soil pH had more influence on AMF colonization than other factors (host plant, phosphorous and C/N ratio). Ouzounidou et al. [58] reported that mycorrhizal colonization of *Salvia hispanica* L. (*Lamiaceae*) tended to increase at a pH level above 7. At pH ranging between 5.5 and 7, mycorrhizal colonization rate did not vary significantly for spring oats (*Poaceae*) [59]. AMF are commonly found under near neutral to alkaline soil pH [60]. The increase of mycorrhizal colonization with pH could be explained by the presence of a well-adapted AMF community, having great ability to colonize host plant roots even in alkaline soil [61].

Concerning soil organic nitrogen (ON) contents, which varied between 0.32 and 1.48 mg g^-1^, they were considered as low to medium [62]. Mycorrhizal root colonization decreased with increasing soil ON. Under low nitrogen concentrations in the soil, plant-fungi competition for nitrogen increased, resulting in less mycorrhizal growth. Only when the nitrogen demand of the fungus was satisfied, the mycorrhizal growth response became positive [63, 64]. It has been demonstrated that N concentration in AMF mycelium was higher (5%), as compared to the plant shoots and roots (≤ 1%) [65]. This could be explained by the substantial nitrogen demand of AMF for the synthesis of protein and chitin, the main constituents of their cell walls [66, 67].

Our data showed that the available phosphorus amounts were between 18.7 and 107 ppm in the different studied sites, which can be considered as middle to high soil P levels according to Holford and Cullis [68]. According to the PLS-path model, mycorrhizal colonization was significantly and inversely affected by high P levels in the soil of the different studied sites. This result is in accordance with previous studies, which reported that high P availability induced low AMF colonization rates [69, 70]. When P is abundant, a symbiotic relationship may become superfluous and the host plant does not need to invest in AMF [51].

### Mycorrhizal colonization and climatic variables

Among all environmental variables, we found that mycorrhizal root colonization of barley was more related to climatic variables. Our results demonstrated that the average annual rainfall that was between 324–508 mm in the different sites had a significant and inverse effect on mycorrhization of barley. The higher percentage of AMF root colonization was related to the lower rainfall. Several previous studies are in accordance with our results [71, 32]. Low precipitation generally decreased soil humidity and increased oxygen concentrations, resulting in AMF spore germination and growth [72]. Zhang et al. [20] showed that rainfall was one of the most influential factors that directly affected the hyphal length density of AMF. However, it has been shown in other studies that increased precipitation was associated with enhanced AMF colonization [73, 74]. These divergent results could be explained by the difference in other environmental conditions such as temperature, soil texture and evaporation. That is why in this study PLS-SEM as a multivariate approach was used to understand these complex relationships between climate, soil physico-chemical properties and AMF root colonization.

The effect of climate was supported by a direct correlation between mycorrhizal colonization and the maximum temperature of the warmest month that ranged between 26.2 and 29.3°C across the studied sites. These findings were in line with those of Frater et al. [75], studying AMF symbiosis in different geographic locations having the same temperature range as our sites (24–28°C). The response curve of AMF colonization to temperature is generally unimodal, i.e. when it exceeded an optimum it had a negative effect [31]. Increased temperature seems to enhance root elongation rate, leading to a better AMF root colonization [75]. At a low temperature, nutrient acquisition by AMF is reduced leading to a decrease in mycorrhizal colonization [76].

In contrast, AMF root colonization of barley was inversely affected by altitude, which in the present study ranged between 2 and 649 m a.s.l. Temperature decreases with increasing altitude [77], which limits plant growth by inhibiting their nutrient absorption and water uptake. The relationship between mycorrhizal colonization and altitude varied significantly according to the host plant species [78]. This could be due to the species dependency on the mycorrhizal symbiosis or to its vegetation density [79].

Taken together, our findings showed that soil properties and climatic characteristics influence mycorrhizal colonization of *H*. *vulgare* roots in the sampled sites. The PLS-path model revealed that climatic factors and chemical soil properties explained a moderate (*R*^2^ = 39%) part of AMF colonization variation of barley. At a large scale, Soudzilovskaia et al. [31] found that both soil fertility in terms of pH, soil C and N availability and site climate, accounted for 50% (*R*^2^) of the variability in AMF colonization of vascular plants. Using a canonical analysis approach, Posada et al. [80] reported that the influence of environmental and physico-chemical soil variables explained 37% of the variation of mycorrhizal root colonization of *Brachiaria decumbens* (*Poaceae*). Similarly, Yang et al. [81], using a structural equation model analysis, showed that environmental factors (aridity, plant biomass, soil organic carbon, total nitrogen and pH) accounted for 56% of the variability of root length colonization in Tibetan grassland.

In the present study, mycorrhizal colonization of barley was directly influenced by temperature and inversely by rainfall. Most of the studies examining the effect of climate on AMF colonization were conducted in the greenhouse and laboratory conditions [82, 83]. AMF could tolerate a wide temperature range. These fungi had the ability to produce trehalose, which protects cells under stress conditions such us heat [15]. However, the effect of temperature on mycorrhizal differs among ecotypic differentiation [84] and AMF taxa [82]. Changes in precipitation could also affect AMF communities [85, 86]. In fact, the optimum temperature or precipitation for AMF growth differ among AMF species [87]. Compared to laboratory experiments, only few field studies were conducted [88, 89]. In the present study, the PLS-SEM demonstrated that from all climate variables only the direct effects of temperature and rainfall on mycorrhization of barley were significant, whereas their indirect effects through soil properties were weak and not significant. Therefore, these indirect effects were deleted from the final path model to avoid any confusion. In agricultural ecosystems, indigenous AMF species supposed to be more tolerant to stressful conditions [90, 91] can therefore, be used to enhance plant performance under future climate scenarios.

## Conclusions

The current research showed that physical soil properties had no significant influence on AMF root colonization of barley in Northern Tunisia. However, chemical soil properties and climatic characteristics were the main environmental factors influencing mycorrhizal root colonization. The PLS path model method demonstrated that climate characteristics have more relevance on barley mycorrhization than chemical soil properties. Therefore, our findings demonstrated that this model can be used to understand the response of AMF to other environmental conditions in different Tunisian ecosystems.

## Supporting information

S1 Fig Decomposition of total inertia. (TIF)

S2 Fig Dendrogram. (TIF)

S1 Table One-way ANOVA of the effect of the different sites on the variation of AMF root colonization of barley. (DOCX)

S2 Table Relative matrix with quantitative dissimilarities. (DOCX)

S3 Table VIF values of manifest variables. (DOCX)
