# Supplementary figures and images for: Higher temperatures and lower annual rainfall do not restrict, directly or indirectly, the mycorrhizal colonization of barley (Hordeum vulgare L.) under rainfed conditions
Source: PLoS One. 2020 Nov 5;15(11):e0241794. doi: 10.1371/journal.pone.0241794 (PMC7644023; doi:10.1371/journal.pone.0241794)

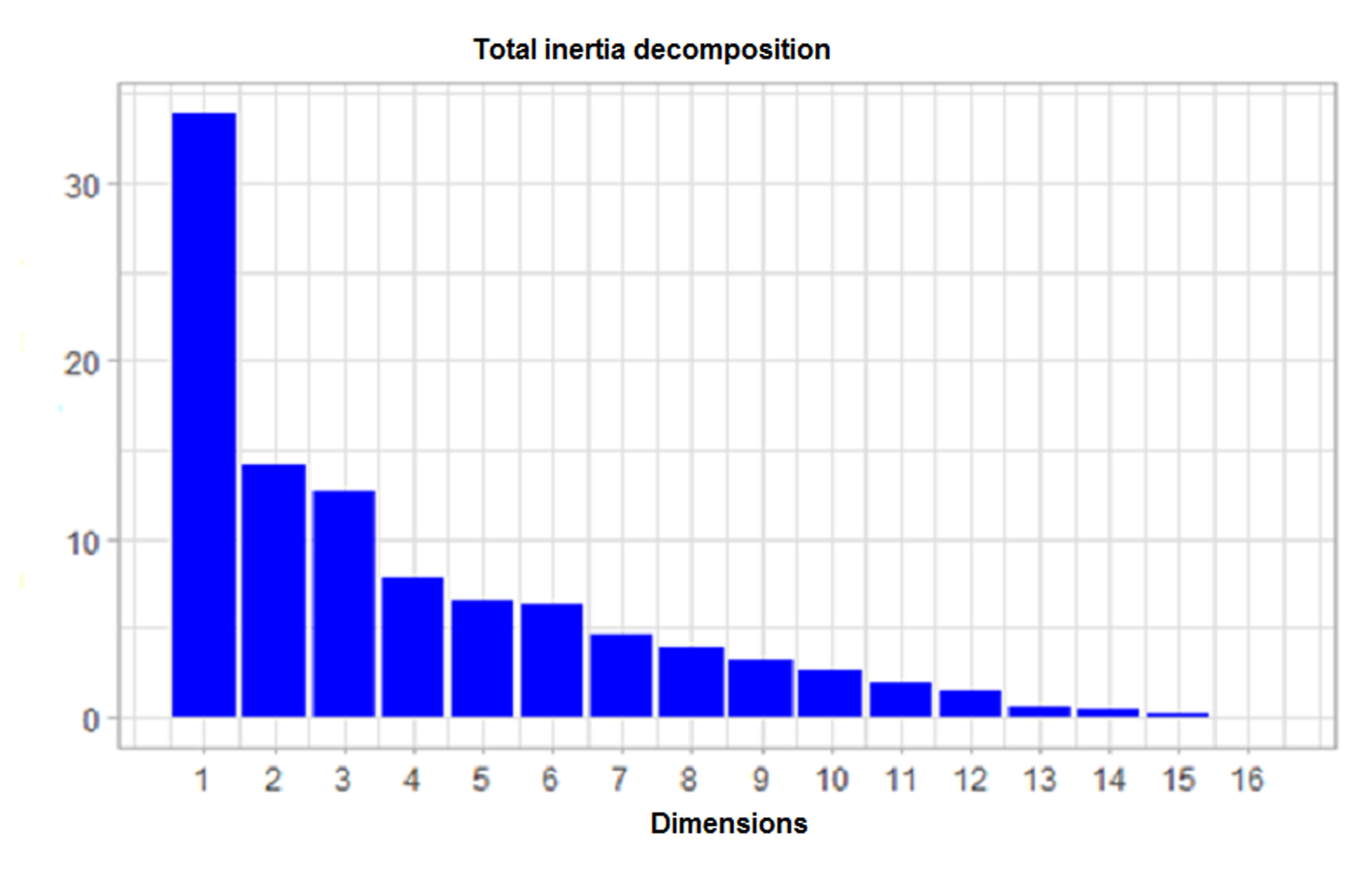

Supplement: S1 Fig — (TIF) [file pone.0241794.s001.tif]

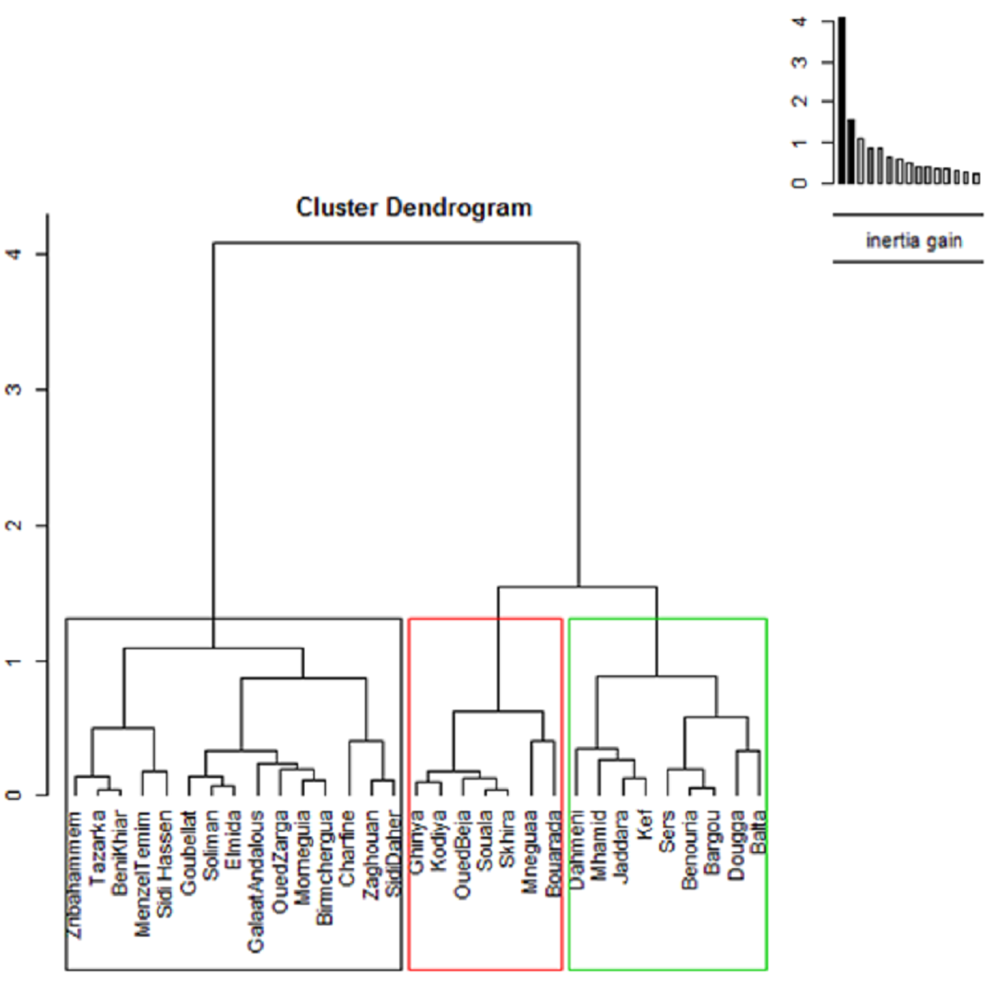

Supplement: S2 Fig — (TIF) [file pone.0241794.s002.tif]
