# Supplementary material for: Higher temperatures and lower annual rainfall do not restrict, directly or indirectly, the mycorrhizal colonization of barley (Hordeum vulgare L.) under rainfed conditions
Source: PLoS One. 2020 Nov 5;15(11):e0241794. doi: 10.1371/journal.pone.0241794 (PMC7644023; doi:10.1371/journal.pone.0241794)
Supplement: S1 Table — (DOCX) [file pone.0241794.s003.docx]

**S1 Table.**

| Source of variation | df | Mean of square | *F* values |
| --- | --- | --- | --- |
| Mycorrhizal rate | 30 | 643.7 | 38.7*** |
| Residual | 62 | 16.6 |  |
| Arbuscules rate | 30 | 541.3 | 35.47*** |
| Residual | 62 | 15.3 |  |
| Vesicles rate | 30 | 33.68 | 6.11*** |
| Residual | 62 | 5.52 |  |
