# Supplementary material for: Higher temperatures and lower annual rainfall do not restrict, directly or indirectly, the mycorrhizal colonization of barley (Hordeum vulgare L.) under rainfed conditions
Source: PLoS One. 2020 Nov 5;15(11):e0241794. doi: 10.1371/journal.pone.0241794 (PMC7644023; doi:10.1371/journal.pone.0241794)
Supplement: S2 Table — (DOCX) [file pone.0241794.s004.docx]

**S2 Table.**

| **Cluster 1**  **Variable** | v. test | Mean in category | Overall mean | Sd in category | Overall sd | *p*-values |
| --- | --- | --- | --- | --- | --- | --- |
| pH | 2.5729 | 7.9576 | 7.7184 | 0.3621 | 0.3776 | 0.0101 |
| OC | -2.4362 | 0.7848 | 1.0043 | 0.2564 | 0.3659 | 0.0148 |
| ON | -3.0649 | 0.3361 | 0.6381 | 0.0431 | 0.4002 | 0.0022 |
| CaCO_3_ | -2.3786 | 18.5455 | 29.5183 | 12.3721 | 18.7387 | 0.0174 |
| SSC | 2.7443 | 41.2000 | 30.1871 | 14.4742 | 16.3007 | 0.0061 |
| SMC | -1.9818 | 9.8818 | 12.3935 | 4.2878 | 5.1483 | 0.0475 |
| AAR | -4.4348 | 343.2364 | 413.1935 | 12.2700 | 64.0760 | 0.0000 |
| M | 2.8615 | 28.2455 | 27.6806 | 0.5945 | 0.8018 | 0.0042 |
| m | 3.9949 | 10.5182 | 8.6839 | 0.9907 | 1.8651 | 0.0001 |
| Z | -3.1895 | 66.0909 | 215.5806 | 60.3527 | 190.3865 | 0.0014 |
| MC | 3.5843 | 39.9303 | 27.2151 | 15.1352 | 14.4098 | 0.0003 |
| Arb | 3.5486 | 38.1152 | 26.5710 | 13.4177 | 13.2143 | 0.0004 |
| **Cluster 2**  **Variable** | v. test | Mean in category | Overall mean | Sd in category | Overall sd | *p*-values |
| SCC | 3.9291 | 50.8667 | 37.7355 | 9.4599 | 14.5473 | 0,0001 |
| SSC | -2.9940 | 18.9750 | 30.1871 | 12.3552 | 16.3007 | 0,0028 |
| Ves | -2.2682 | 0.3694 | 2.0871 | 1.2253 | 3.2963 | 0,0233 |
| **Cluster3**  **Variable** | v. test | Mean in category | Overall mean | Sd in category | Overall sd | *p*-values |
| ON | 3.7273 | 1.0999 | 0.6381 | 0.2282 | 0.4002 | 0.0002 |
| CaCO_3_ | 2.4074 | 43.4833 | 29.5183 | 16.7439 | 18.7387 | 0.0161 |
| SCC | -3.1194 | 23.6875 | 37.7355 | 9.6568 | 14.5473 | 0.0018 |
| SMC | 2.0903 | 15.7250 | 12.3935 | 5.8485 | 5.1483 | 0.0366 |
| AAR | 3.0762 | 474.2125 | 413.1935 | 20.5233 | 64.0760 | 0.0021 |
| Z | 4.5585 | 484.2500 | 215.5806 | 109.6799 | 190.3865 | 0.0000 |
| M | -3.4977 | 26.8125 | 27.6806 | 0.3655 | 0.8018 | 0.0005 |
| m | -3.7174 | 6.5375 | 8.6839 | 0.8985 | 1.8651 | 0.0002 |
| MC | -2.0219 | 18.1958 | 27.2151 | 4.5582 | 14.4098 | 0.0432 |
| Arb | -2.0473 | 18.1958 | 26.5710 | 4.5582 | 13.2143 | 0.0406 |
