# Supplementary material for: Higher temperatures and lower annual rainfall do not restrict, directly or indirectly, the mycorrhizal colonization of barley (Hordeum vulgare L.) under rainfed conditions
Source: PLoS One. 2020 Nov 5;15(11):e0241794. doi: 10.1371/journal.pone.0241794 (PMC7644023; doi:10.1371/journal.pone.0241794)
Supplement: S3 Table — (DOCX) [file pone.0241794.s005.docx]

**S3 Table.**

| Manifest variables | First VIF values | Final VIF values |
| --- | --- | --- |
| pH | 1.239 | 1.239 |
| Salinity | 1.266 | 1.266 |
| OC | 1.619 | 1.541 |
| ON | 1.563 | 1.320 |
| P | 1.258 | 1.117 |
| Total CaCO_3_ | 5.783 | - |
| SCC | 2.567 | 2.567 |
| SSC | 2.935 | 2.935 |
| SMC | 1.739 | 1.739 |
| AAR | 3.232 | 2.361 |
| M | 2.453 | 2.130 |
| m | 5.783 | - |
| Z | 3.509 | 2.140 |
| Total AMF root colonization rate | 1.000 | 1.000 |
